# Supplementary material for: Global regulation via modulation of ribosome pausing by the ABC-F protein EttA
Source: Nat Commun. 2024 Jul 26;15:6314. doi: 10.1038/s41467-024-50627-z (PMC11282234; doi:10.1038/s41467-024-50627-z)
Supplement: Supplementary file 1 — Supplementary information [file 41467_2024_50627_MOESM1_ESM.pdf]

Supplementary information

Global regulation via modulation of ribosome pausing by the ABC-F protein EttA

Farès Ousalem[1§], Saravuth Ngo[1], Thomas Oïffer[1], Amin Omairi-Nasser[1], Marion Hamon[2], Laura Monlezun[1], Grégory Boël[1,*]

[1] Expression Génétique Microbienne, CNRS, Université Paris Cité, Institut de Biologie Physico-Chimique, F-75005 Paris, France. [2] CNRS, Institut de Biologie Physico-Chimique, Plateforme de Protéomique, FR550, F-75005 Paris, France.

[§] Current addresses: Biomarqueurs et nouvelles cibles thérapeutiques en oncologie, INSERM U981, Université Paris Saclay, Institut de Cancérologie Gustave Roussy, 11 rue Edouard Vaillant, 94805 Villejuif Cedex, France.

[*]Correspondence to:
Grégory Boël, Institut de Biologie Physico-Chimique, 13 rue Pierre et Marie Curie, 75005 Paris, France, Tel: +33-1-58415121; e-mail: boel@ibpc.fr

Contents:

Supplementary Figures 1 to 8

Supplementary Tables 1 to 7

Supplementary References

Supplementary Fig. 1

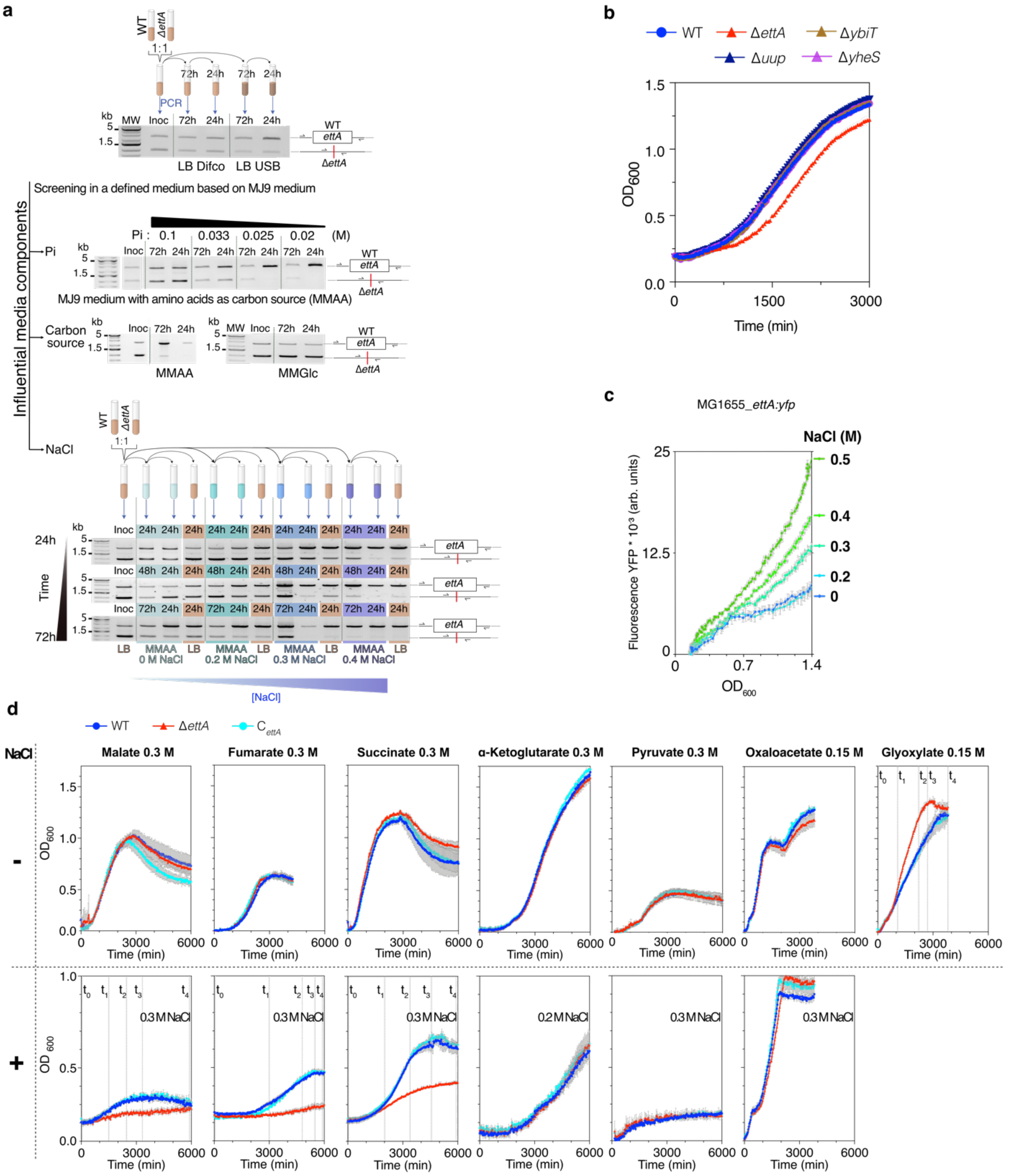

Supplementary Fig. 1: Phenotypic characterization of culture conditions*.* a, Agarose gels showing PCR products from the *ettA* locus in the inoculum, co-culture for 72 h (or 48 h and 24 h in the lower panel) and sub-cultured cultures (+ 24 h) to evaluate the proportion of each strain during fitness experiments between the WT and *ΔettA* strains. Firstly, fitness assay in LB media from two different suppliers: Difco Becton Dickinson and USB-Affymetrix. Secondly from the top, fitness assay in the MJ9 medium[1], with 16 aa as carbon source at 250 mM (glycine at 26.6 mM, alanine at 22.4 mM, valine at 17 mM, leucine at 15.2 mM, isoleucine at 15.2 mM, proline at 17.3 mM, phenylalanine at 12.1 mM, serine at 19 mM, threonine at 16.8 mM, asparagine at 15.1 mM, glutamine 13.7 mM, aspartic acid at 15 mM, glutamic acid at 13.6 mM, arginine at 11.4 mM, histidine at 12.8 mM and lysine at 13.6 mM) where the phosphate concentration has been adjusted to different concentrations (0.1, 0.033, 0.025 and 0.02 M) for 72 hours. Thirdly, fitness in MMAA medium (same as above with 25 mM

phosphate) and MMGlc medium where Glucose at 22 mM is used as carbon source in place of the aa. Finally, fitness assays at 24, 48 and 72 hours (from top to bottom) in MMAA medium and in MMAA medium supplemented with increasing concentrations of NaCl (0.2, 0.3 and 0.4 M; left to right). **b**, Graph showing the growth of the WT strain and strains deleted for one of the 4 ABC-F paralogs (Δ*ettA,* Δ*yheS,* Δ*ybiT* and Δ*uup*) in MMAA 0.4 M NaCl culture medium. Error bars represent mean ± standard deviation (s.d.) for triplicate experiments. **c,** Fluorescence of cell cultures expressing *ettA* gene in translational fusion with a *yfp* gene in the MMAA medium in the presence of NaCl at different concentrations (0, 0.2, 0.3, 0.4 and 0.5 M). Curves represent the mean and the standard deviation of the YFP fluorescence at the corresponding OD_{600} during the growth of three independent cultures. **d,** Effect of salt stress on the growth of the WT, Δ*ettA* and C_{ettA} strains in minimum medium (MM) with different intermediate metabolites of the TCA cycle as carbon sources. The OD_{600} was measured every 30 minutes on three independent cultures using a plate reader. Error bars represent mean ± s.d. for triplicate experiments. The time points marked on certain growth curves correspond to the OD values reported in **Fig. 1c**.

Supplementary Fig. 2

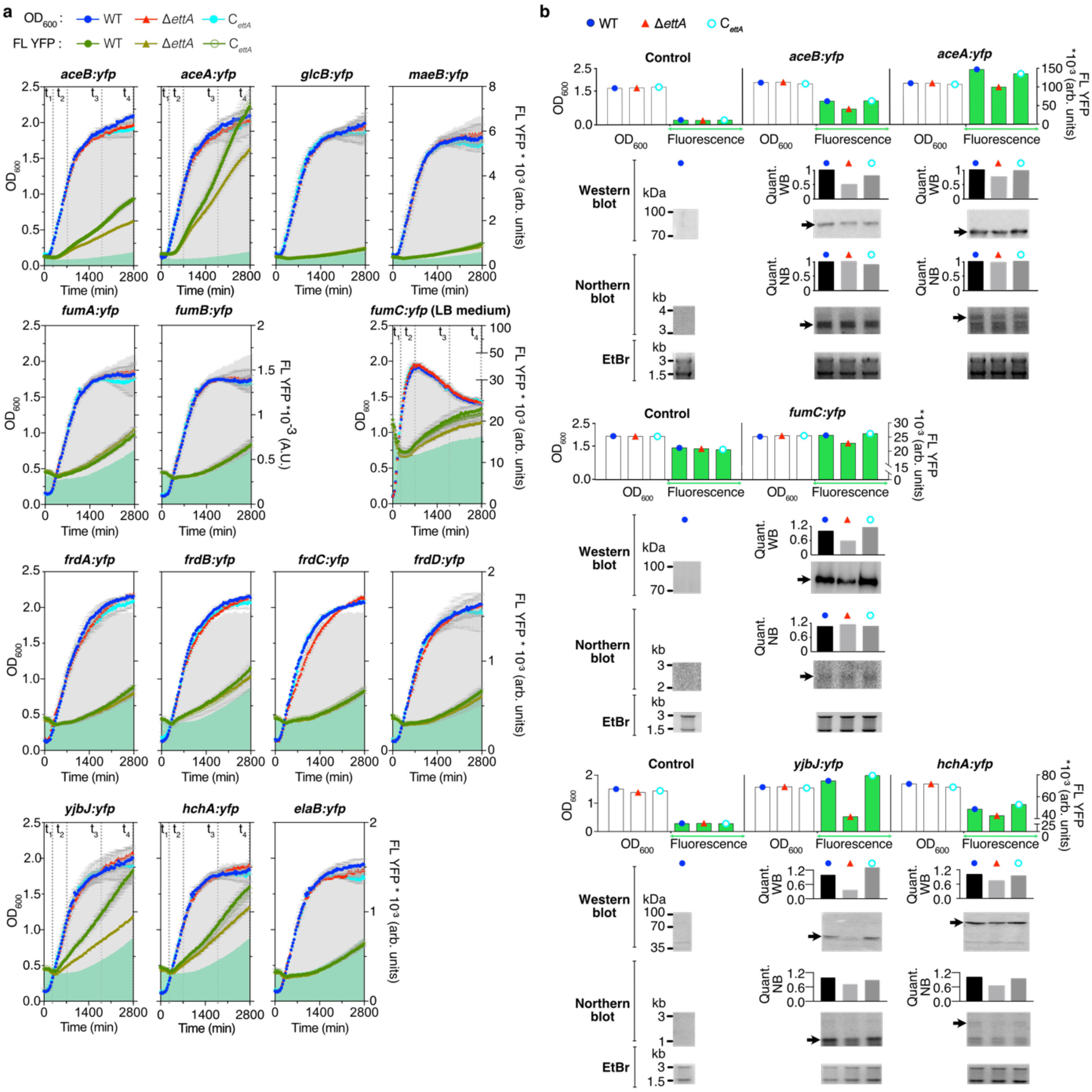

Supplementary Fig. 2: Validation of the change in expression of some genes in the Δ*ettA* strain with chromosomal *yfp* reporter fusions. a, Growth curves (blue, cyan and red) and fluorescence emission curves (green gradient) of triplicate cultures of the WT, Δ*ettA* and C_{ettA} strains expressing the *yfp* gene fused to genes involved in the TCA cycle (*aceB:yfp, aceA:yfp, glcB:yfp, maeB:yfp, fumA/B:yfp, frdA/B/C/D:yfp*) and to genes with other functions (*yjbJ:yfp, hchA:yfp* and *elaB:yfp*). Growth was in MMAA medium except for *fumC:yfp* in LB medium. OD_{600} and fluorescence measurements were made using a ClarioStar microplate reader every 30 min. The gray cloud in the graph corresponds to the growth of the WT control strain without a fusion under the same culture condition. The green cloud in the graph corresponds to the residual fluorescence of the WT control strain without YFP fusion. Error bars represent mean ± s.d. for triplicate experiments. The time points marked on certain growth curves correspond to the fluorescence values reported in **Fig. 2f**. **b,** Validation of the EttA-induced deregulation for *aceB/A:yfp*, *fumC:yfp*, *yjbJ:yfp* and *hchA:yfp* genes by western blot and northern blot. Strains with *yfp* fusions to the different targets were grown in MMAA medium except for *fumC:yfp* in LB medium. Top, measurements of the OD_{600} and fluorescence after 48 h of culture in MMAA medium. Control shows background fluorescence from the WT, Δ*ettA* and C_{ettA} strains. Middle, Western-blot with anti-GFP on protein extracts from the same cultures. Bottom, Northern blot on total RNA extracted from the same cultures, detected with a radioactive probe hybridizing to the *yfp* gene. Bands signals were quantified using Fiji

Supplementary Fig. 2 (cont.)

software as described in the methods section. Gels stained with Ethidium Bromide are shown at the bottom of the blots as loading control.

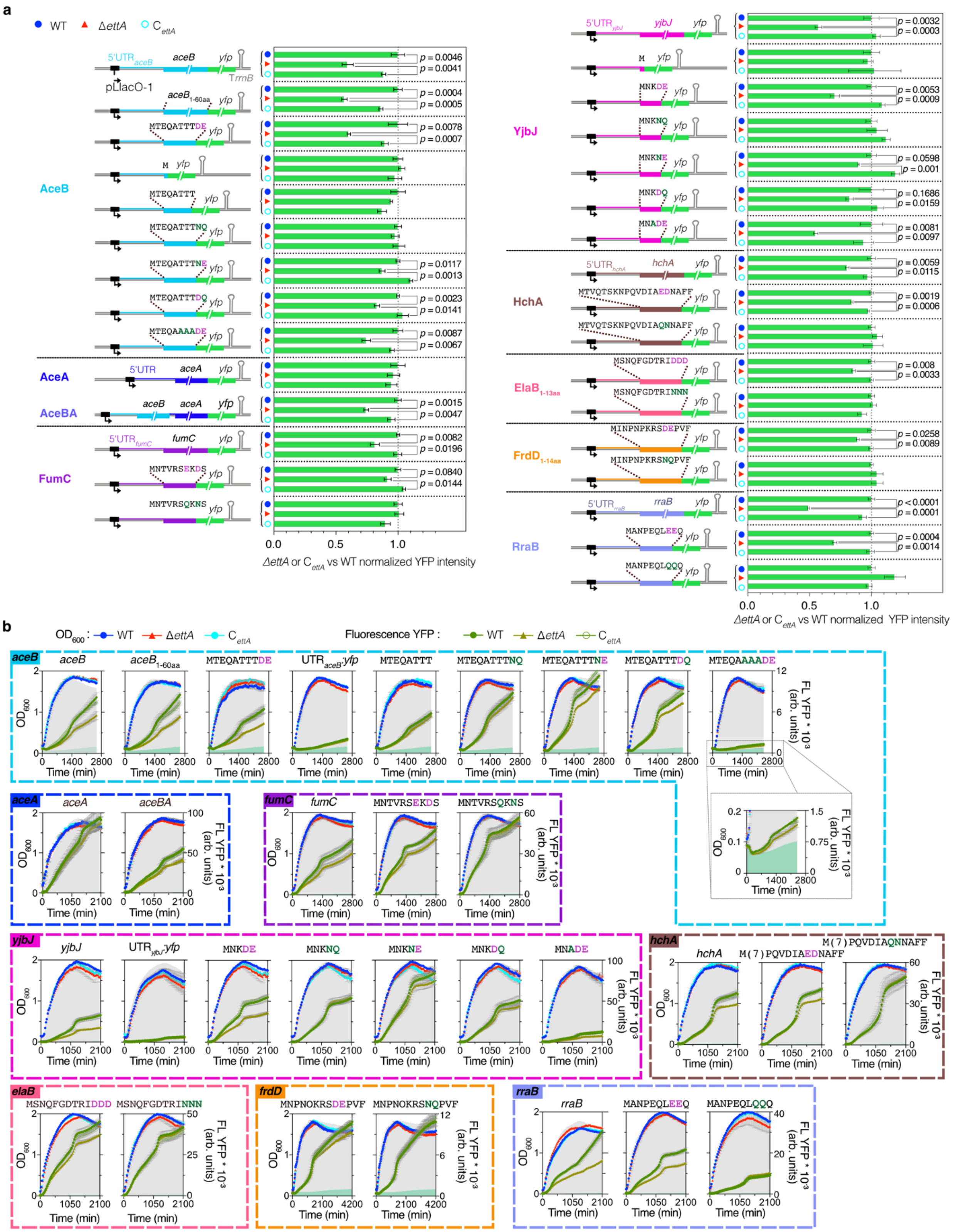

Supplementary Fig. 3: EttA rescues translation of mRNAs with sequences harboring a motif of two consecutive acidic residues. a, Histograms showing the ratios of YFP intensity in the WT, Δ*ettA* or C_{ettA} strains normalized over the WT strain (see methods) representing the expression of genes (*aceB*, *aceA*, *fumC*, *yjbJ*, *hchA*, *elaB, frdD* and *rraB*) in fusion with a *yfp* gene. The various truncated and mutated forms of each fusion tested are shown. All the constructs conserved the 5'UTR sequences of the different genes tested and are expressed from a pMMB plasmid. The tested constructs are shown on the left side of the histograms.

Values used to calculate the ratios for all the targets correspond to those at the end of the growth curves presented in panel (**b**). Error bars represent mean ± s.d. for triplicate experiments. For each target, the relative fluorescence value of the WT strain is equal to 1. The *p* values were determined by unpaired two-tailed *t*-tests. **b,** Growth curves of the three strains (WT, Δ*ettA* and C_{ettA}) with the different plasmid constructs tested in panel (**a**). Cultures were grown in LB_Amp100 medium in the presence of 1 mM IPTG. OD_{600} and fluorescence measurements were made as described in **Supplementary Fig. 2a.** Error bars represent mean ± s.d. for triplicate experiments. All the growth curves corresponding to different versions of the same target gene are presented within a colored frame corresponding to the color used in panel (**a**): *aceB* (cyan), *aceA* (blue), *fumC* (purple), *yjbJ* (magenta), *hchA* (brown), *elaB* (pink), *frdD* (orange), and *rraB* (light blue).

Supplementary Fig. 4

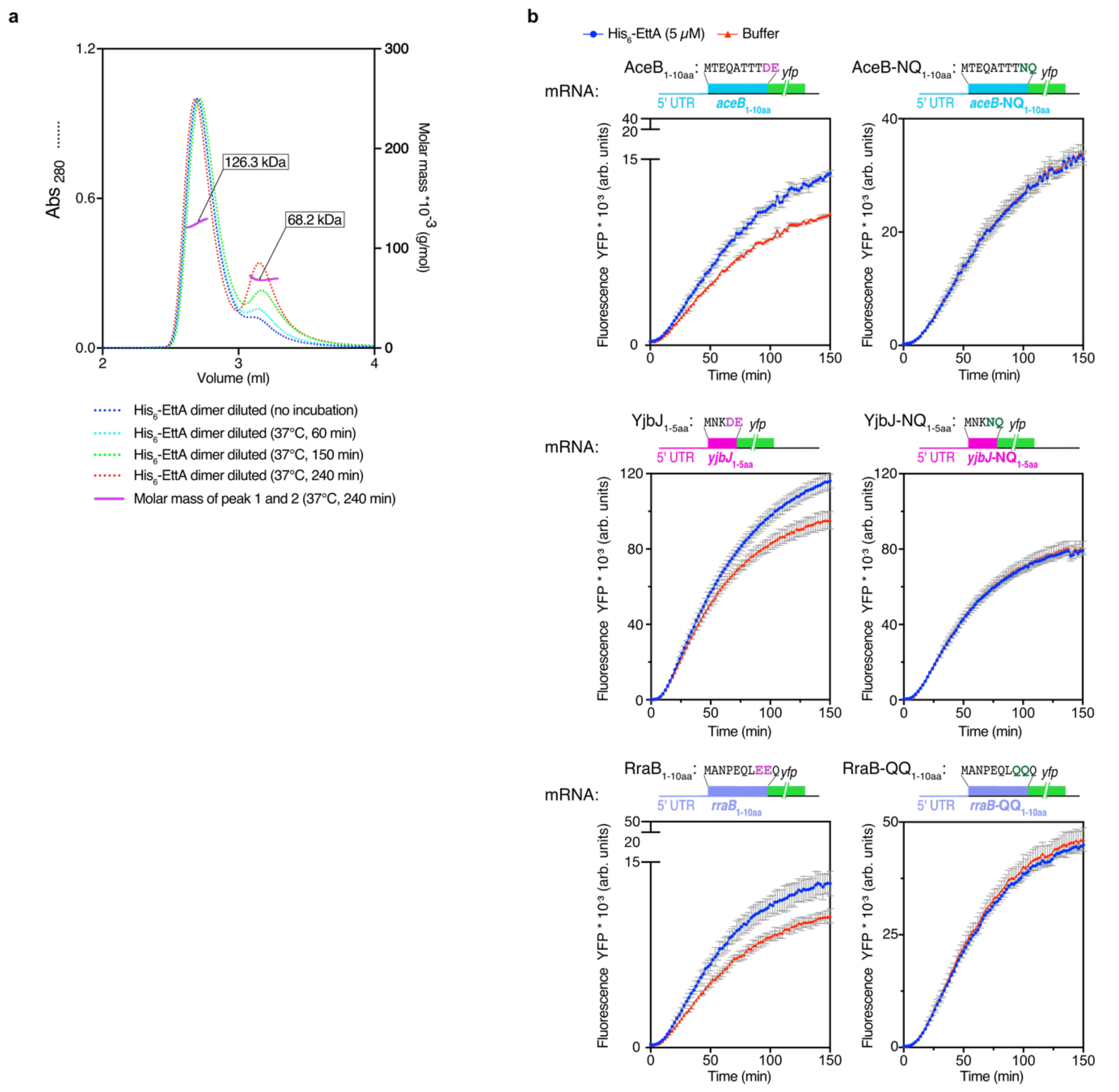

Supplementary Fig. 4: *In vitro* validation of the involvement of EttA in the regulation of the translation of certain mRNAs. **a,** Size exclusion chromatography-multiangle light scattering (SEC-MALS) analysis of the His$_6$-EttA protein purified from *E. coli.* The purified His$_6$-EttA protein at 675 µM was diluted to 40 µM in the same buffer as that used during the purification but with 5% glycerol and heated at 37 °C for 60 min (light blue dotted line), 150 min (green dotted line), or 240 min (red dotted line) before injection. The unheated protein is shown with a blue dotted line. The dotted lines correspond to the absorbance measurements at 280 nm (left axis) and the solid magenta line to the molecular weights (right axis) as a function of the elution volume. The first peak corresponds to His$_6$-EttA protein in its dimeric form (estimated at 126.3 kDa) and the second peak corresponds to the monomeric form (estimated at 68.2 kDa). **b,** Curves showing YFP intensity of *in vitro* translation products from purified mRNAs expressing truncated targets (*aceB*$_{1\text{-}10aa}$, *yjbJ*$_{1\text{-}5aa}$, *rraB*$_{1\text{-}10aa}$) fused with a *yfp* reporter gene, with mutations in acidic residues (NQ or QQ) or without (DE or EE), in the presence (blue) or absence (red) of His$_6$-EttA at 5 µM. Translation reactions were performed with the PURExpress ΔRibosome kit and purified ribosomes from the *E. coli* MRE600 in a final volume of 10 µl for 150 min at 37 °C using a microplate reader.

Supplementary Fig. 5

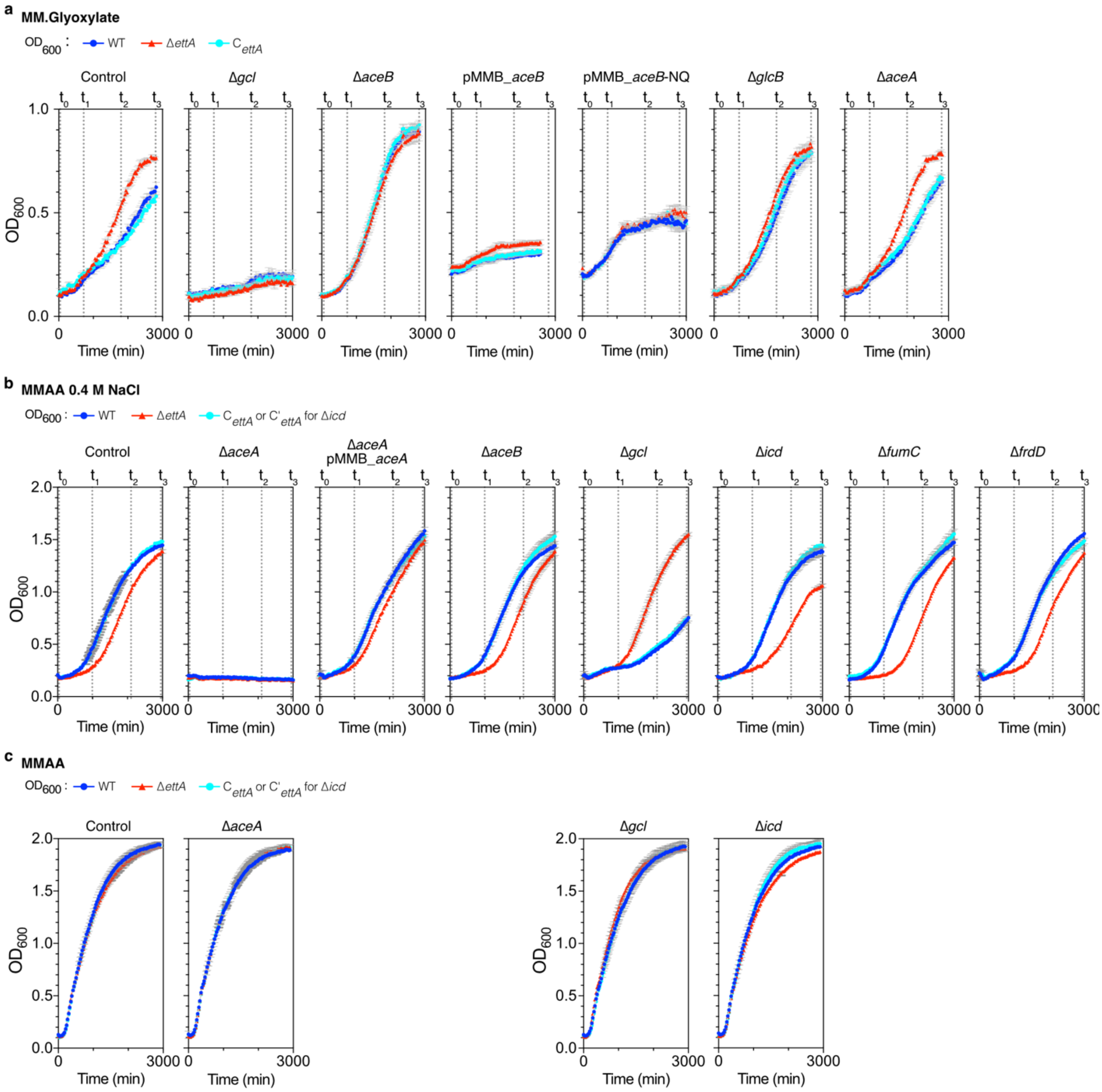

Supplementary Fig. 5: Growth of the WT, Δ*ettA* and C$_{ettA}$ strains carrying deletions in the genes involved in the metabolism of TCA cycle carbon intermediates in MM.Glyoxylate medium or in MMAA medium in the presence or absence of NaCl. a, The three strains (WT, Δ*ettA* and C$_{ettA}$) with deletions of the *glc* (Δ*gcl*), *aceB* (Δ*aceB*), *glcB* (Δ*glcB*) or *aceA* (Δ*aceA*) genes or with plasmids over-expressing *aceB* or *aceB*-NQ were grown in MM.Glyoxylate. **b,** The three strains (WT, Δ*ettA* and C$_{ettA}$) with deletions of the *aceB* (Δ*aceB*), *aceA* (Δ*aceA*), *fumC* (Δ*fumC*), *frdD* (Δ*frdD*), *icd* (Δ*icd*) or *gcl* (Δ*gcl*) genes or with a plasmid overexpressing *aceA* were grown in MMAA 0.4 M NaCl **c,** The three strains (WT, Δ*ettA* and C$_{ettA}$) with deletions in the *aceA* (Δ*aceA*), *gcl* (Δ*gcl*) or *icd* (Δ*icd*) genes, were grown in MMAA without NaCl. **a, b and c,** Growth curves were obtained as described in **Supplementary Fig. 1d.** The OD$_{600}$ was measured every 30 minutes on three independent cultures using a plate reader. Error bars represent mean ± s.d. for triplicate experiments. Amp100 and 1 mM IPTG were added to cultures for strains with the pMMB_*aceB*, pMMB_*aceB*-NQ or pMMB_*aceA* plasmids. The time points marked on some growth curves correspond to the OD values reported in **Fig. 4b and c**.

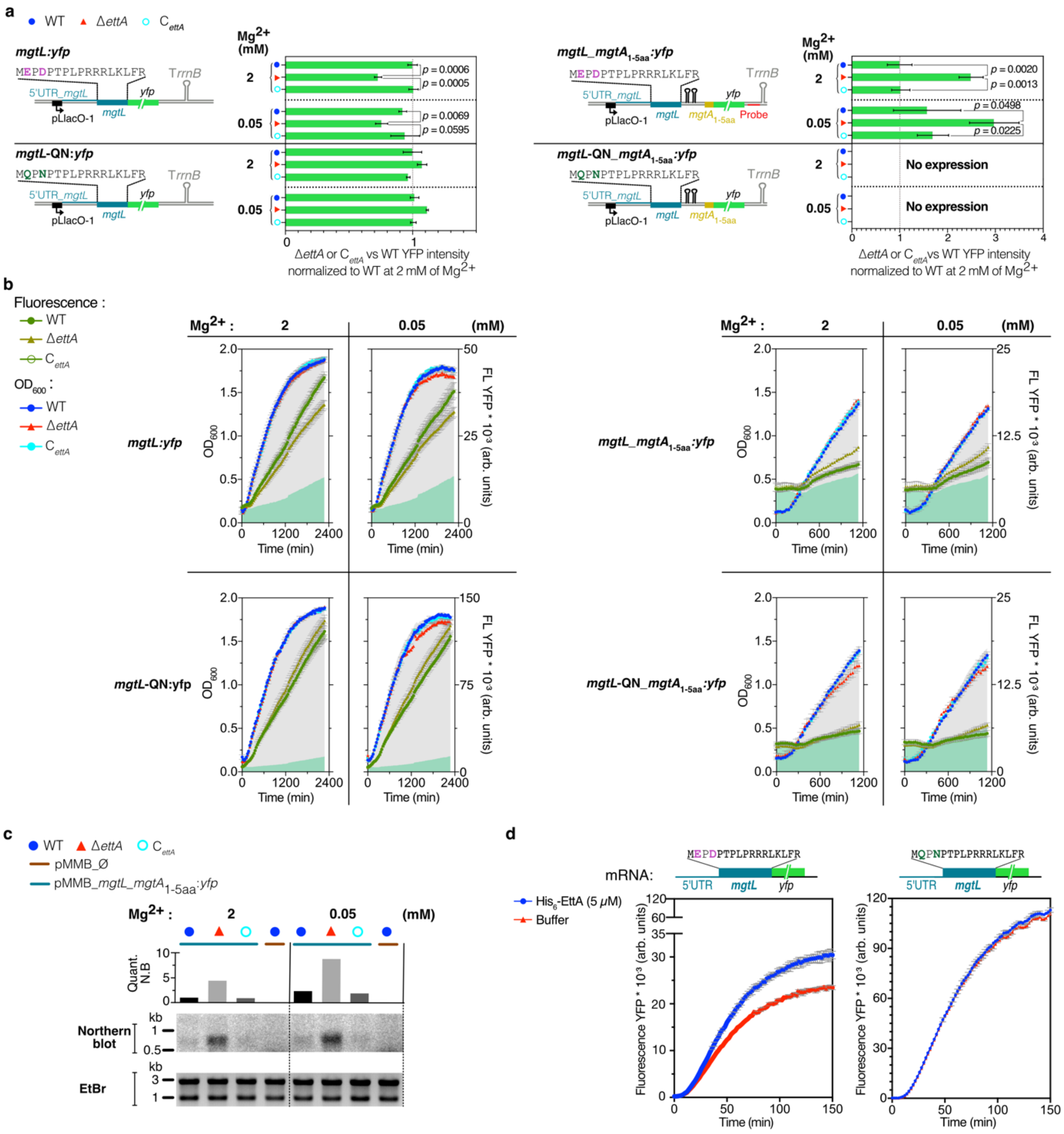

Supplementary Fig. 6: EttA partly controls the regulation of the leader peptide MgtL. a, Histograms showing the ratios of YFP intensity in the WT, Δ*ettA* or C*ettA* strains normalized over the fluorescence of WT strain in the 2 mM Mg^{2+} condition (see Methods), for the *mgtL* (*mgtL:yfp*, left) or for *mgtL* with the 5 first codons of *mgtA* (*mgtL_mgtA*1-5aa*:yfp*, right) fused to a *yfp* gene. The strains were grown in MMAA medium with different concentrations of Mg^{2+} (2 and 0.05 mM) and Amp100. Mutated forms of the same constructs *mgtL*-QN*:yfp* (left, bottom) and *mgtL*-QN_*mgtA*1-5aa*:yfp* (right, bottom) where the Glu2 and Asp4 of MgtL are replaced by Gln and Asn respectively are also shown. All the constructs conserved the 5'UTR sequences of the different genes tested and are expressed on an IPTG-inducible pMMB plasmid. The expression ratios for all cultures are calculated from values at the end of the growth curves presented in panel (**b**). For *mgtL:yfp* and *mgtL*-QN:*yfp, mgtL_mgtA*1-5aa*:yfp* and *mgtL*-QN_*mgtA*1- 5aa*:yfp*, the fluorescence value in the WT strain at 2 mM of Mg^{2+} is considered equal to 1 and the values of Δ*ettA* or C*ettA* strains for each condition are normalized to this value. Ratios are calculated at t=2280 min and t=1140 min for the left and right histograms respectively. All the tested constructs are represented on the left side of the histograms. Error bars correspond to mean ± s.d. for triplicate experiments. The *p* values of unpaired two-tailed

t-tests are shown on the side of the bars. **b,** Growth curves of the three strains (WT, Δ*ettA* and C_{ettA}) with the different constructs shown in panel (**a**). Cultures were performed in MMAA medium at different Mg^{2+} concentrations (2 and 0.05 mM) in the presence of 1 mM IPTG and Amp100. Growth curves were obtained by measuring the OD_{600} every 30 min using a plate reader. OD_{600} and fluorescence measurements were performed as described in **Supplementary Fig. 2a.** Error bars represent mean ± s.d. for triplicate experiments. **c,** Northern blot analysis of *mgtL_mgtA*$_{1\text{-}5aa}$*:yfp* transcript using the oligonucleotide probe for the 3'UTR of the *yfp* (see panel **a**, right). RNAs were extracted after 1140 min of growth from the cultures shown in panel (**b**). Expression of *mgtL_mgtA*$_{1\text{-}5aa}$*:yfp* in the presence of Mg^{2+} at different concentrations (2 and 0.05 mM) was measured for the 3 strains (WT, Δ*ettA* and C_{ettA}) and a quantification of the bands is presented in the histogram above the Northern Blot. Staining of the gel with Ethidium Bromide is shown at the bottom of the blot as loading control. **d,** *In vitro* translation of the purified *mgtL:yfp* reporter mRNA in its WT or mutated form (*mgtL*$_{QN}$*:yfp*) and in the presence (blue) or absence (red) of His_6-EttA protein at 5 µM as described in **Supplementary Fig. 4b**. Error bars represent mean ± s.d. for triplicate experiments.

Supplementary Fig. 7

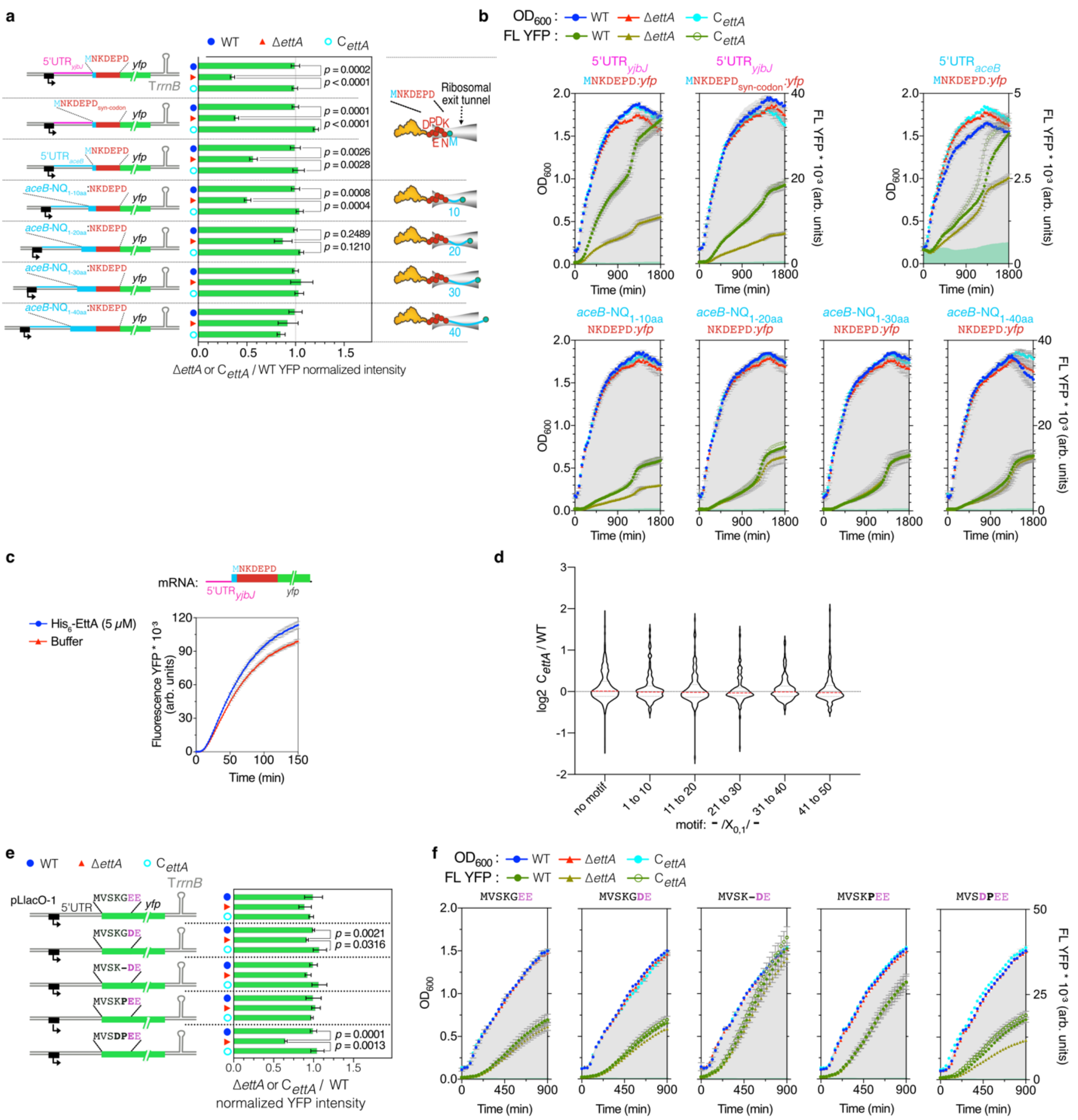

Supplementary Fig. 7: EttA is a translation factor acting as a facilitator of the synthesis of peptide sequences having the DE or DXE motif in the first 20 amino acids. a, Histogram showing the translation efficiency of a *yfp* reporter mRNA according to the localization of the NKDEPD motif within the first 40 neosynthesized amino acids, in the three strains (WT, Δ*ettA* and C_{ettA}). Translation efficiency is calculated as the ratio of Δ*ettA* / WT or C_{ettA} / WT ratios of YFP intensities. Seven modified constructs were tested: three express the *yfp* gene with a N-terminal MNKDEPD sequence preceded by either the 5'UTR of *aceB* or the 5'UTR of *yjbJ* or the 5'UTR of *yjbJ* followed by a MNKDEPD sequence expressed from an RNA using synonymous aa codons of the MNKDEPD motif (syn-codon, see methods). The other four constructs express the *yfp* gene in fusion with the NKDEPD motif placed either after 10 aa (M(10)NKDEPD), 20 aa (M(20)NKDEPD), 30 aa (M(30)NKDEPD) or 40 aa (M(40)NKDEPD) after the initiation codon, the inserted sequence used for the motif displacement was the *aceB*-NQ. Fluorescence measurements were taken after 1800 min of culture in LB_Amp100 medium with 1 mM IPTG using a microplate reader. Error bars represent mean ± s.d. for triplicate experiments. **b**, Growth curves of the three strains (WT, Δ*ettA* and C_{ettA}) with the different plasmid constructs tested in panel (**a**). OD_{600} and fluorescence measurements were carried out as described in **Supplementary Fig. 2a**.

Supplementary Fig. 7 (cont.)

Bacteria were grown in LB_Amp100 medium in the presence of 1 mM IPTG. Error bars represent mean ± s.d. for triplicate experiments. **c,** *In vitro* translation of a *yfp* reporter mRNA fused to a 5'UTR$_{yjbJ}$-MNKDEPD sequence, in the presence (blue) or absence (red) of His$_6$-EttA protein at 5 µM as described in **Supplementary Fig. 4b**. Error bars represent mean ± s.d. for triplicate experiments. **d,** Violin plot showing the expression level of genes containing the E/D-E/D or E/D-X-E/D motif (- /X$_{0,1}$/ -) or not within the first 50 neo-synthesized aa by windows of 10 aa based on the log$_2$ Δ*ettA*/ C$_{ettA}$ ratios obtained in the S$_{15}$ proteomic. The plot show genes expression distributions over n = 476 for absence of the motif, n = 116 for motif between residues 1 to 10, n = 200 for motif between residues 11 to 20, n = 176 for motif between residues 21 to 30, n = 177 for motif between residues 31 to 40 and n = 150 for motif between residues 41 to 50. The red dotted line is the median and the grey ones the quartiles, p values were calculated using a two-tailed Mann-Whitney test and were all above 0.05. **e,** Histograms showing the ratios of YFP intensity in Δ*ettA* or C$_{ettA}$ strains vs. WT strain of mutants of the first N-terminal residues of the YFP sequence. The tested constructs are presented on the left side of the histograms. All the constructs have the 5'UTR sequence of the original plasmid pMMBp_EH67-*yfp*. Values used to calculate the ratios for all the constructs correspond to those obtained at t=900 min in the panel (**f**). For each target, the fluorescence value of the WT strain is considered equal to 1. Error bars represent mean ± s.d. for triplicate experiments. **a and e**, The p values of unpaired two-tailed t-tests are shown on the side of the bars. **f,** Growth curves of the three strains (WT, Δ*ettA* and C$_{ettA}$) with the different plasmid constructs shown in panel (**e**). OD$_{600}$ and fluorescence measurements were performed as described in **Supplementary Fig. 2a**. Error bars represent mean ± s.d. for triplicate experiments. Cultures were performed in LB_Amp100 medium in the presence of 1 mM IPTG.

Supplementary Fig. 8

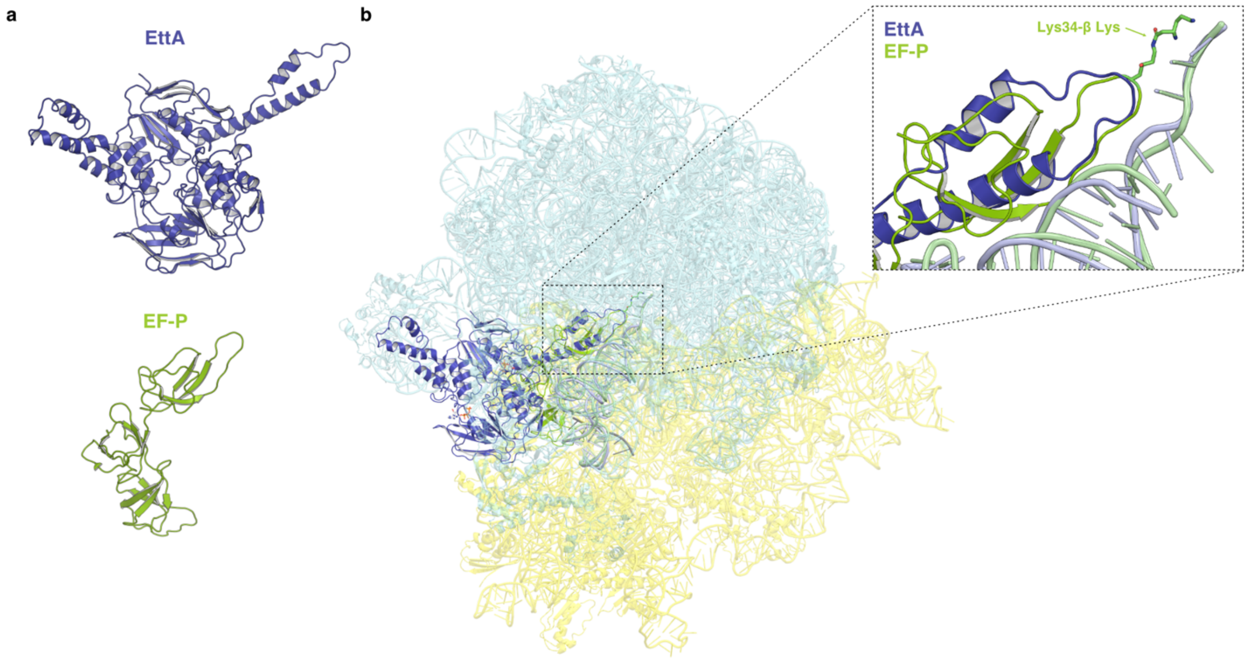

Supplementary Fig. 8: Structural homology between EttA and EF-P extension toward the PTC. a, Structure of EttA-EQ_2 (dark blue) and EF-P isolated from the structure of ribosomal complex (EMD-29398)[2] and (6ENJ)[3] respectively. **b,** Structures of EttA-EQ_2 (dark blue) in complex with 70S IC complex (EMD-29398)[2] aligned on the domain V of the 23S rRNA of the structure (6ENJ)[3] of the Polyproline-stalled ribosome in complex with EF-P (green). The 50S (cyan) and 30S (yellow) subunits of the EttA-EQ_2 ribosomal complex are presented in transparency, a zoomed view of the extension of EF-P and the extremity of the PtIM of EttA toward the PTC that interact with the CCA acceptor stem of the P-site tRNA is presented, top right.

Supplementary Tables

Supplementary Table 1. Reference strains

Strains	Genotype/ Parent strain	Method used for strain construction	References
DH5α	F⁻ *endA1 glnV44 thi-1 recA1 relA1 gyrA96 deoR nupG purB20* φ80d*lacZ*ΔM15 Δ(*lacZYA-argF*)U169, hsdR17($r_K^- m_K^+$), λ⁻	-	[4]
MRE600	F- rna	-	[5]
MG1655	K-12 F⁻ λ⁻ *ilvG⁻ rfb-50 rph-1*	-	[6]
Δ*ettA*	MG1655 Δ*ettA*	-	[7]
Δ*uup*	MG1655 Δ*uup*		
Δ*ybiT*	MG1655 Δ*ybiT*		
Δ*yheS*	MG1655 Δ*yheS*		
C_{ettA}	MG1655 Δ*ettA, P21:ettA*	pOSIP_CT (P21) plasmid[8]	This study
C'_{ettA}	MG1655 Δ*ettA, attB:ettA*	pOSIP_KL (*attB*) plasmid[8]	This study

Supplementary Table 2. Strains with genomic YFP reporter

Strain name	Method used for strains construction	Selection
MG1655_*aceA:yfp*	P1 transduction from Venus-YFP (GFP) fusion strains[9] to parental strain MG1655, Δ*ettA* or C_{ettA}	Cm^R
Δ*ettA_aceA:yfp*		
C_{ettA}_*aceA:yfp*		
MG1655_*aceB:yfp*		
Δ*ettA_aceB:yfp*		
C_{ettA}_*aceB:yfp*		
MG1655_*glcB:yfp*		
Δ*ettA_glcB:yfp*		
C_{ettA}_*glcB:yfp*		
MG1655_*fumA:yfp*		
Δ*ettA_fumA:yfp*		
C_{ettA}_*fumA:yfp*		
MG1655_*fumB:yfp*		
Δ*ettA_fumB:yfp*		
C_{ettA}_*fumB:yfp*		
MG1655_*fumC:yfp*		
Δ*ettA_fumC:yfp*		
C_{ettA}_*fumC:yfp*		
MG1655_*frdA:yfp*		
Δ*ettA_frdA:yfp*		
C_{ettA}_*frdA:yfp*		
MG1655_*frdB:yfp*		
Δ*ettA_frdB:yfp*		
C_{ettA}_*frdB:yfp*		
MG1655_*frdC:yfp*		
Δ*ettA_frdC:yfp*		
C_{ettA}_*frdC:yfp*		
MG1655_*frdD:yfp*		
Δ*ettA_frdD:yfp*		
C_{ettA}_*frdD:yfp*		
MG1655_*elaB:yfp*		
Δ*ettA_elaB:yfp*		
C_{ettA}_*elaB:yfp*		
MG1655_*maeB:yfp*		
Δ*ettA_maeB:yfp*		
C_{ettA}_*maeB:yfp*		
MG1655_*yjbJ:yfp*		
Δ*ettA_yjbJ:yfp*	The sequence of the *yfp* and Cm^R was PCR amplified from the *aceB:yfp* strain DNA by primers with a 60 nucleotides homology of upstream and downstream of the stop codon of target gene. Genomic integration was performed using lambda-Red recombineering then the construction was transduced into the Δ*ettA* and C_{ettA} strains (See methods).	
C_{ettA}_*yjbJ:yfp*		
MG1655_*hchA:yfp*		
Δ*ettA_hchA:yfp*		
C_{ettA}_*hchA:yfp*		

Supplementary Table 3. Deletion strains constructed in this study

Strain name	Method used for strain construction	Selection
MG1655_Δ*aceA*	P1 transduction from Keio collection deletion strain[10] to parental strain MG1655, Δ*ettA* or C_{ettA} (C'_{ettA} for Δ*icd*)	Kan^R
Δ*ettA*_Δ*aceA*		
C_{ettA}_Δ*aceA*		
MG1655_Δ*glcB*		
Δ*ettA*_Δ*glcB*		
C_{ettA}_Δ*glcB*		
MG1655_Δ*fumC*		
Δ*ettA*_Δ*fumC*		
C_{ettA}_Δ*fumC*		
MG1655_Δ*frdD*		
Δ*ettA*_Δ*frdD*		
C_{ettA}_Δ*frdD*		
MG1655_Δ*gcl*		
Δ*ettA*_Δ*gcl*		
C_{ettA}_Δ*gcl*		
MG1655_Δ*icd*		
Δ*ettA*_Δ*icd*		
C'_{ettA}_Δ*icd*		

Supplementary Table 4. Plasmids constructed during this work

Plasmids	Selection	References
pKD46	Amp^R	11
pMMBpLacO-1-67EH-*yfp*	Amp^R	12
pMMB-Ø (pMMB-67EH)	Amp^R	13
pMMB-*aceB*:*yfp*	Amp^R	This study
pMMB_UTR$_{aceB}$:*yfp*	Amp^R	This study
pMMB-*aceB*$_{1\text{-}60aa}$:*yfp*	Amp^R	This study
pMMB-*aceB*$_{1\text{-}10aa}$:*yfp*	Amp^R	This study
pMMB-*aceB*$_{1\text{-}8aa}$:*yfp*	Amp^R	This study
pMMB-*aceB*-NQ$_{1\text{-}10aa}$:*yfp*	Amp^R	This study
pMMB-*aceB*-D9N$_{1\text{-}10aa}$:*yfp*	Amp^R	This study
pMMB-*aceB*-E10Q$_{1\text{-}10aa}$:*yfp*	Amp^R	This study
pMMB-*aceB*-TTT/AAA$_{1\text{-}10aa}$:*yfp*	Amp^R	This study
pMMB-*aceB*-NQ:*yfp*	Amp^R	This study
pMMB-*fumC*:*yfp*	Amp^R	This study
pMMB-*fumC* $_{1\text{-}10aa}$:*yfp*	Amp^R	This study
pMMB_UTR-*fumC*-QN$_{1\text{-}10aa}$:*yfp*	Amp^R	This study
pMMB-*rraB*:*yfp*	Amp^R	This study
pMMB-*rraB*$_{1\text{-}10aa}$:*yfp*	Amp^R	This study
pMMB-*rraB*-QQ$_{1\text{-}10aa}$:*yfp*	Amp^R	This study
pMMB-*yjbJ*:*yfp*	Amp^R	This study
pMMB-*yjbJ*$_{1\text{-}5aa}$:*yfp*	Amp^R	This study
pMMB-*yjbJ*-NQ$_{1\text{-}5aa}$:*yfp*	Amp^R	This study
pMMB-*yjbJ*-D4N$_{1\text{-}5aa}$:*yfp*	Amp^R	This study
pMMB-*yjbJ*-E5Q$_{1\text{-}5aa}$:*yfp*	Amp^R	This study
pMMB-*yjbJ*-K3A$_{1\text{-}5aa}$:*yfp*	Amp^R	This study
pMMB_UTR$_{yjbJ}$:*yfp*	Amp^R	This study
pMMB-*hchA*:*yfp*	Amp^R	This study
pMMB-*hchA*$_{1\text{-}20aa}$:*yfp*	Amp^R	This study
pMMB-*hchA*-QN$_{1\text{-}20aa}$:*yfp*	Amp^R	This study
pMMB-*frdD*$_{1\text{-}14aa}$:*yfp*	Amp^R	This study
pMMB-*frdD*-NQ$_{1\text{-}14aa}$:*yfp*	Amp^R	This study
pMMB-*elaB*$_{1\text{-}15aa}$:*yfp*	Amp^R	This study
pMMB-*elaB*-NNN$_{1\text{-}15aa}$:*yfp*	Amp^R	This study
pMMB-*aceA*:*yfp*	Amp^R	This study
pMMB-*aceB*-*aceA*:*yfp*	Amp^R	This study
pMMB-*aceB*	Amp^R	This study
pMMB-*aceB*-NQ	Amp^R	This study
pMMB-*aceA*	Amp^R	This study
pMMB-*mgtL*_*mgtA*$_{1\text{-}5aa}$:*yfp*	Amp^R	This study
pMMB-*mgtL*-QN_*mgtA*$_{1\text{-}5aa}$:*yfp*	Amp^R	This study
pMMB-*mgtL*:*yfp*	Amp^R	This study
pMMB-*mgtL*-QN:*yfp*	Amp^R	This study
pMMB_UTR$_{aceB}$-MNKDEPD:*yfp*	Amp^R	This study
pMMB_UTR$_{yjbJ}$-MNKDEPD:*yfp*	Amp^R	This study
pMMB_UTR$_{yjbJ}$-MNKDEPD$_{syn\text{-}codon}$:*yfp*	Amp^R	This study
pMMB_UTR$_{aceB}$_aceB-NQ$_{1\text{-}10aa}$:NKDEPD: *yfp*	Amp^R	This study
pMMB_UTR$_{aceB}$_aceB-NQ$_{1\text{-}20aa}$:NKDEPD: *yfp*	Amp^R	This study
pMMB_UTR$_{aceB}$_aceB-NQ$_{1\text{-}30aa}$:NKDEPD: *yfp*	Amp^R	This study
pMMB_UTR$_{aceB}$_aceB-NQ$_{1\text{-}40aa}$:NKDEPD: *yfp*	Amp^R	This study
pMMB-*yfp*$_A$	Amp^R	14
pMMB_UTR$_{aceB}$_*yfp*-MVSKGDE	Amp^R	This study
pMMB_UTR$_{aceB}$_*yfp*-MVSK-DE	Amp^R	This study
pMMB_UTR$_{aceB}$_*yfp*-MVSKPDE	Amp^R	This study
pMMB_UTR$_{aceB}$_*yfp*-MVSDPEE	Amp^R	This study
pBAD-Ø (pBAD/myc-His A)	Amp^R	Invitrogen
pBAD-*His*$_6$-*ettA*	Amp^R	15
pBAD-*His*$_6$-*ettA*_EQ$_2$	Amp^R	15

Supplementary Table 5. Oligonucleotides pairs used for genomic constructions and validation.

No.	Name	Sequence (5'→3')	PCR template	Purpose
L41	EttA_448up-R	CGCAAAGAGTTCCAGAACTTGC	Bacteria	Fitness assays
L42	EttA_439dwn-F	GATCATCGGTGATCTGGCGG		
F01	EttA-pOSIP-P21-F	GAATTCGAGCTCGGTACCCGGCTTTTACTGCGAGGGTGATC	MG1655 genomic DNA	Genomic complementation of *ettA* gene at P21 locus
F02	EttA-pOSIP-P21-R	CATGCATCTCGAGGCATGCCATTTTACGCATTACTTCGC		
F03	EttA-pOSIP-attB-F	GAATTCGAGCTCGGTACCCGGCTTTTACTGCGAGGGTGATCG		Genomic complementation of *ettA* gene at lambda locus (*attB*)
F04	EttA-pOSIP-attB-R	CATGCATCTCGAGGCATGCCATTTTACGCATTACTTCGC		
F05	Primer FLIP_F	ATCTGGTGCTGGGTCTGGTG	Strain to validate	Used to verify the integration of the *ettA* gene on the genome
F06	P4 P21(T)	TAGAACTACCACCTGACC		
F07	P1 P21(T)	ATCGCCTGTATGAACCTG		
F08	P4 Lambda (L)	TCTGGTCTGGTAGCAATG		
F09	P3 Lambda (L)	GGGAATTAATTCTTGAAGACG		
F010	P2 Lambda (L)	ACTTAACGGCTGACATGG		
F011	P1 Lambda (L)	GGCATCACGGCAATATAC		
E027	fusion-yjbJ-yfp-F	CAGAAAGATCAGGCAGAAAAAGAGGTCGTGGATTGGGAAACCCGCAATGAATATCGCTGGACTAGTGCGGCCGCG	Venus yfp (*gfp)* library[9] genomic DNA	Strain constructions with fluorescent *yfp* reporter
E028	fusion-yjbJ-yfp-R	GAAAAAGAAACCGCGTTAAGAGGGCGTACATCCTTGTACACGTCGGGCAGGAGGGATTAATGTAGGCTGGAGCTGCTTCG		
E031	fusion-hchA-yfp-F	TTTGCAGCGAATGCGTTGGGTAAACTGGCGGCGCAGGAAATGCTGGCAGCTTACGCGGGTACTAGTGCGGCCGCG		
E032	fusion-hchA-yfp-R	CGTCGTGAGTACTAACGCGGCCGCGATTGATTATGCGCTTACATTCAAACGTAACAGGGATGTAGGCTGGAGCTGCTTCG		
E029	fusion-elaB-yfp-F	CAAGGAATTGGTGTGGGCGCGGCCGTTGGGCTGGTACTAGGACTGTTGCTGGCACGCCGTACTAGTGCGGCCGCG		
E030	fusion-elaB-yfp-R	AAAGCCTCACATTATACGGGGTACTACAAAAAAATGCAGTACCCCGGTGTAGGGAGGTTTGTAGGCTGGAGCTGCTTCG		
F018	yjbJ-dwn-F	ACTGGCGCAATGGTACCTGG	Strain to validate	Verification of the integration of the *yfp* fusion and gene deletion
F019	yjbJ-up-R	TTGAAGCACATGGGCTCTGTG		
F024	hchA-F -200nt	CACTTGCGACGACGTTGCTC		
F025	hchA-R +200nt	ATTGCCGAATTGCATGGGGG		
F022	elaB-F -200nt	TCACTGGCCTGATAAGCCTG		
F023	elaB-R +220nt	TTTGCTGACTGGCCAGCCAG		
D057	aceB-F	GTTGTGCTGAACGAAAAGAGC		
F081	aceB-R up	CTTCAATACCCGCTTTCGCC		
D056	aceA-F	GGAACAGATCACCACTTCCG		
F080	aceA-R dwn	GTCGGCCTGTTCGAAACG C		
D062	glcB-F	GGGCGTTTCTGGTTTAACCG		
F083	glcB-R	TTACAGCAACAGTACGCCGC		
G033	gcl-F	TAATGTCTGTCGCATCCCGC		
G034	gcl-R	CGCAGCTTTCTCAAAACGGG		
G041	icd-F	GCCGCATTATAGCCTAATAACG		
G042	icd-R	ACCCCAAAACTACCGAGGGG		
D059	maeB-F	AGTCTGTAGACTCCGGCAG		Verification of the integration of the *yfp* fusion
E052	YFP_intern-R	GTGGCGGATCTTGAAGTTGG		
D046	fumA-F	CGCTTTTAACAGGGCAACGG		
E052	YFP_intern-R	GTGGCGGATCTTGAAGTTGG		
D047	fumB-F	AAATGCACTTTGCGTGCCGC		
E052	YFP_intern-R	GTGGCGGATCTTGAAGTTGG		
D048	fumC-F	ATAAACAGAGCCGCCCTTCG		
E052	YFP_intern-R	GTGGCGGATCTTGAAGTTGG		
D049	frdA-F	GAAGATTACTACGCTGCCGC		
E052	YFP_intern-R	GTGGCGGATCTTGAAGTTGG		
D050	frdB-F	GCCAGCTAAACGCGTTTACG		
E052	YFP_intern-R	GTGGCGGATCTTGAAGTTGG		
D052	frdC-F	AAACACGTCGATCCGGCTG		
E052	YFP_intern-R	GTGGCGGATCTTGAAGTTGG		
D053	frdD-F	TGGGACCAGAGCCAATTATC		
E052	YFP_intern-R	GTGGCGGATCTTGAAGTTGG		
D048	fumC-F	ATAAACAGAGCCGCCCTTCG		Verification of the gene deletion
J262	fumC-R	GTGCCGGACGTTGGTGAGTT		
D053	frdD-F	TGGGACCAGAGCCAATTATC		
J263	frdD-R	CAAATGTGGAGCAAGAGGCG		

Supplementary Table 6. Oligonucleotide pairs used for pMMB plasmid constructions.

No.	Name	Sequence (5'→3')	PCR template	Purpose
G029	start_yfp-F	GTGAGCAAGGGCGAGGAG	pMMBpLlacO-1-67EH-*yfp*	pMMB backbone
G072	pMMB_pLac-R	TGTGCTCAGTATCTTGTTATCCGC		
H009	utr_aceB_pLac-F	ATAACAAGATACTGAGCACAGTGCTGAACGAAAAGAGCAC	MG1655 genomic DNA	Insert for pMMB-*aceB:yfp*
H010	aceB_pLac-R	AGCTCCTCGCCCTTGCTCACCGCTAACAGGCGGTAGCC		
H023	60AA_aceB-yfp-R	GCTCCTCGCCCTTGCTCACGTTATCAATATCTTGCTGCTGCTG	pMMB-*aceB:yfp*	Construction pMMB-*aceB*$_{1-60aa}$*:yfp*
G029	start_yfp-F	GTGAGCAAGGGCGAGGAG		
H021	10AA_aceB-yfp-R	CTCCTCGCCCTTGCTCACTTCATCGGTTGTTGTTGCCTG	pMMB-*aceB:yfp*	Construction pMMB-*aceB*$_{1-10aa}$*:yfp*
G029	start_yfp-F	GTGAGCAAGGGCGAGGAG		
H054	10AA-D9N_E10Q_aceB-yfp-R	CTCGCCCTTGCTCACTTGATTGGTTGTTGTTGCCTGTTC	pMMB-*aceB:yfp*	Construction pMMB-*aceB*-NQ$_{1-10aa}$*: yfp*
G029	start_yfp-F	GTGAGCAAGGGCGAGGAG		
I063	10AA_D9N_aceB-yfp-R	TCCTCGCCCTTGCTCACTTCATTGGTTGTTGTTGCCTGTTCAGTC	pMMB-*aceB*$_{1-10aa}$*:yfp*	Construction pMMB-*aceB*-D9N$_{1-10aa}$*:yfp*
G029	start_yfp-F	GTGAGCAAGGGCGAGGAG		
I064	10AA_E10Q_aceB-yfp-R	TCCTCGCCCTTGCTCACTTGATCGGTTGTTGTTGCCTGTTCAGTC	pMMB-*aceB*$_{1-10aa}$*:yfp*	Construction pMMB-*aceB*-E10Q$_{1-10aa}$*: yfp*
G029	start_yfp-F	GTGAGCAAGGGCGAGGAG		
H055	10AA_TTT/AAA_aceB-yfp-R	CTCGCCCTTGCTCACTTCATCGGCTGCTGCTGCCTGTTCAGTCATCGTGC	pMMB-*aceB*$_{1-10aa}$*:yfp*	Construction pMMB-*aceB*-TTT/AAA $_{1-10aa}$*:yfp*
G029	start_yfp-F	GTGAGCAAGGGCGAGGAG		
H047	8AA_aceB-yfp-R	CTCGCCCTTGCTCACGGTTGTTGTTGCCTGTTCAGTC	pMMB-*aceB*$_{1-8aa}$*:yfp*	Construction pMMB-*aceB*$_{1-8aa}$*:yfp*
G029	start_yfp-F	GTGAGCAAGGGCGAGGAG		
H053	5AA_aceB-yfp-R	TGAACAGCTCCTCGCCCTTGCTCACTGCCTGTTCAGTCATCGTGC	pMMB-*aceB*$_{1-5aa}$*:yfp*	Construction pMMB-*aceB*$_{1-5aa}$*:yfp*
G029	start_yfp-F	GTGAGCAAGGGCGAGGAG		
H034	utr_aceB-0aa_pLac-R	CTCCTCGCCCTTGCTCATCGTGCAGCTCCTCGTC	pMMB-*aceB:yfp*	Construction pMMB_UTR$_{aceB}$*;yfp*
G029	start_yfp-F	GTGAGCAAGGGCGAGGAG		
I014	fumC_pMMB-F	ATAACAAGATACTGAGCACAGTGAGCTAAAAGTTGCTTAACGAAAG	MG1655 genomic DNA	Insert for pMMB-*fumC:yfp*
I013	fumC_pMMB-R	AGCTCCTCGCCCTTGCTCACACGCCCGGCTTTCATACTGC		
I016	10aa_fumC-R	AGCTCCTCGCCCTTGCTCACCGAATCTTTTTCGCTGCGTACTG	pMMB-*fumC:yfp*	Construction pMMB-*fumC*$_{1-10}$*:yfp*
G029	start_yfp-F	GTGAGCAAGGGCGAGGAG		
I017	10aa_E7Q_D9N_fumC-R	AGCTCCTCGCCCTTGCTCACCGAATTTTTTTGGCTGCGTACTGTATTC		Construction pMMB-*fumC*-QN$_{1-10}$aa*:yfp*
G029	start_yfp-F	GTGAGCAAGGGCGAGGAG		
I021	utr_frdD_pMMB-F	ATAACAAGATACTGAGCACATGGGCGGTAACTGTGGTTGC	MG1655 genomic DNA	Insert for pMMB-*frdD:yfp*
I022	utr_frdD_pMMB-R	AGCTCCTCGCCCTTGCTCACGATTGTAACGACACCAATCAGC		
I075	14aa_frdD_pMMB-R	CCTCGCCCTTGCTCACGAATACCGGTTCGTCAGAACGC	pMMB-*frdD:yfp*	Construction pMMB-*frdD*$_{1-14aa}$*:yfp*
G029	start_yfp-F	GTGAGCAAGGGCGAGGAG		
I076	14aa_D10N_E11Q_frdD-R	CTCGCCCTTGCTCACGAATACCGGTTGGTTAGAACGCTTTGGATTTGGATTAAT		Construction pMMB-*frdD*-NQ$_{1-14aa}$*:yfp*
G029	start_yfp-F	GTGAGCAAGGGCGAGGAG		
I069	rraB-pMMB-F	ATAACAAGATACTGAGCACAATCGGCAGGACATTAAGAGGAATG	MG1655 genomic DNA	Insert for pMMB-*rraB:yfp*
I070	rraB-pMMB-R	AGCTCCTCGCCCTTGCTCACGTGGCGAACTCCGTCATCGTC		
I050	10aa_rraB-R	TCCTCGCCCTTGCTCACCTGTTCTTCCAGTTGTTCCGGGTTTGC	pMMB-*rraB:yfp*	Construction pMMB-*rraB*$_{1-10aa}$*:yfp*
G029	start_yfp-F	GTGAGCAAGGGCGAGGAG		
J007	10aa_E8Q_E9Q_rraB_pMMB-R	GCCCTTGCTCACCTGTTGTTGCAGTTGTTCCGGGTTTGCCATG		Construction pMMB-*rraB*-QQ$_{1-10aa}$*:yfp*
G029	start_yfp-F	GTGAGCAAGGGCGAGGAG		
I071	utr_yjbJ-F	ATAACAAGATACTGAGCACAGTACTCTCATTCACAACCTAACGATG	MG1655 genomic DNA	Insert for pMMB-*yjbJ:yfp*
I072	utr_yjbJ-R	AGCTCCTCGCCCTTGCTCACCCAGCGATATTCATTGCGGGTTTC		
I080	5aa_yjbJ-R	CCTCGCCCTTGCTCACTTCATCTTTATTCATAATCAAGACCTC	pMMB-*yjbJ:yfp*	Construction pMMB-*yjbJ*$_{1-5aa}$*:yfp*
G029	start_yfp-F	GTGAGCAAGGGCGAGGAG		
I081	5aa_D4N_E5Q_yjbJ-R	CCTCGCCCTTGCTCACTTGATTTTTATTCATAATCAAGACCTCATCGTTAG		Construction pMMB-*yjbJ*-NQ$_{1-5aa}$*:yfp*
G029	start_yfp-F	GTGAGCAAGGGCGAGGAG		

Supplementary Table 6 (continued)

I079	0aa_yjbJ-R	CCTCGCCCTTGCTCACCATAATCAAGACCTCATCGTTAGGTTG	pMMB-*yjbJ:yfp*	Construction pMMB_UTR$_{yjbJ}$:*yfp*
G029	start_yfp-F	GTGAGCAAGGGCGAGGAG		
J031	5aa_D4N_yjbJ-R	TTTTATTCATAATCAAGACCTCATCGTTAG	pMMB-*yjbJ*$_{1\text{-}5aa}$:*yfp*	Construction pMMB-*yjbJ*-D4N$_{1\text{-}5aa}$:*yfp*
J030	5aa_D4N_yjbJ-F	TGATTATGAATAAAAATGAAGTGAGCAAGGGCGAGG		
J068	5aa_E5Q_yjbJ-R	CCTCGCCCTTGCTCACTTGATCTTTATTCATAATCAAGACCTCATC	pMMB-*yjbJ*$_{1\text{-}5aa}$:*yfp*	Construction pMMB-*yjbJ*-E5Q$_{1\text{-}5aa}$:*yfp*
G029	start_yfp-F	GTGAGCAAGGGCGAGGAG		
J067	5aa_K3A_yjbJ-R	CCTCGCCCTTGCTCACTTCATCTGCATTCATAATCAAGACCTCATCGTTAG	pMMB-*yjbJ*$_{1\text{-}5aa}$:*yfp*	Construction pMMB-*yjbJ*-K3A$_{1\text{-}5aa}$:*yfp*
G029	start_yfp-F	GTGAGCAAGGGCGAGGAG		
I012	utr_hchA_pLac-F	ATAACAAGATACTGAGCACAAGCTCAGTCGCAAATATAGTGAC	MG1655 genomic DNA	Insert for pMMB-*hchA:yfp*
G028	utr_hchA_pLac-R	GCTCCTCGCCCTTGCTCACACCCGCGTAAGCTGCCAGCA		
I018	20aa-hchA-yfp-R	GCTCCTCGCCCTTGCTCACGAAGAATGCATTATCTTCAGCAATATC	pMMB-*hchA:yfp*	Construction pMMB-*hchA*$_{1\text{-}20aa}$:*yfp*
G029	start_yfp-F	GTGAGCAAGGGCGAGGAG		
I019	20aa_E13Q_D4N_hchA-R	AGCTCCTCGCCCTTGCTCACGAAGAATGCATTATTTTGAGCAATATCGAC	pMMB-*hchA:yfp*	Construction pMMB-*hchA*-QN$_{1\text{-}20aa}$: *yfp*
G029	start_yfp-F	GTGAGCAAGGGCGAGGAG		
I059	utr_elaB_pMMB-F	ATAACAAGATACTGAGCACAAGGTTTTACGCAAATGGAGAACGAG	MG1655 genomic DNA	Insert for pMMB-*elaB:yfp*
I060	utr_elaB_pMMB-R	AGCTCCTCGCCCTTGCTCACACGGCGTGCCAGCAACAGTC		
I062	15aa_elaB_pMMB-R	TCCTCGCCCTTGCTCACCGTCAGGTCGTCATCGATACGTG	pMMB-*elaB:yfp*	Construction pMMB-*elaB*$_{1\text{-}15aa}$:*yfp*
G029	start_yfp-F	GTGAGCAAGGGCGAGGAG		
J004	15aa_3N_elaB-F	AATAACAACCTGACGGTGAGCAAGGGC	pMMB-*elaB*$_{1\text{-}15aa}$:*yfp*	Construction pMMB-*elaB*-NNN$_{1\text{-}15aa}$: *yfp*
J005	15aa_3N_elaB-R	CTTGCTCACCGTCAGGTTGTTATTGATACGTGTATCACCAAACTGATTAG		
H011	aceA_pMMB-F	GATAACAAGATACTGAGCACAGTAAACCACCACATAACTATGGAG	MG1655 genomic DNA	Insert for pMMB-*aceA:yfp*
H012	aceA_pMMB-R	GCTCCTCGCCCTTGCTCACGAACTGCGATTCTTCAGTGGAG		
H009	utr_aceB_pLac-F	ATAACAAGATACTGAGCACAGTGCTGAACGAAAAGAGCAC	MG1655_*aceA:yfp* genomic DNA	Insert for pMMB-*aceBA:yfp*
H012	aceA_pMMB-R	GCTCCTCGCCCTTGCTCACGAACTGCGATTCTTCAGTGGAG		
H013	aceA_noyfp_pMMB-F	TAATAATTCGAGCTCGGTACCC	pMMB-*aceA:yfp*	pMMB-*aceA*
H014	aceA_noyfp_pMMB-R	GTACCGAGCTCGAATTATTAGAACTGCGATTCTTCAGTGGAG		
H019	utr_aceB_noyfp_pLac-F	CTACCGCCTGTTAGCGTAATAATTCGAGCTCGGTACCCG	pMMB-*aceB:yfp*	Construction pMMB-*aceB*
H020	utr_aceB_noyfp_pLac-R	TTACGCTAACAGGCGGTAGC		
J036	D9N_E10Q_aceB-F	AACTGGCTTTCACAAGGCCGTATG	pMMB-*aceB*	Construction pMMB-*aceB*-NQ
J037	D9N_E10Q_aceB-R	GCCTTGTGAAAGCCAGTTGATTGGTTGTTGTTGCCTGTTCAGTCATC		
G071	pMMB_pLac_yfp-F	CCCTCGTGACCACCCTGGGC	pMMBpLlacO-1-67EH-*yfp*	pMMB backbone for pMMB-*mgtL_mgtA*$_{1\text{-}5aa}$:*yfp*
G072	pMMB_pLac-R	TGTGCTCAGTATCTTGTTATCCGC		
Twist	mgtL_A-5codon_pMMB-pLac	ATAACAAGATACTGAGCACAGAGATGCTACGAATATTATTGGATTCTCCTTATTATTTGCGGCGCTTTTTTCACTTACCGGAGGTTATATGGAACCTGATCCCACGCCTCTCCCTCGACGGAGATTAAAACTTTTCCGGTAAGCCCGTCTTTTCACGGCGTTACCGGATGCGTAAGGCCGTGACGTTTTAACGTCCCTGCTCAGCTTTATTACCTTCAGGTAAGGCTTCGCCACGCCTGAAGACATTTCTGTACTGTTTCAGACAGTGCGGAGGGACTCCTTATGTTTAAAGAAATTTTTTCCAAAATCGTAAAAATCGTGAGCAAGGGCGAGGAGCTGTTCACCGGGGTGGTGCCCATCCTGGTCGAGCTGGACGGCGACGTAAACGGCCACAAGTTCAGCGTGTCCGGCGAGGGCGAGGGCGATGCCACCTACGGCAAGCTGACCCTGAAGCTGATCTGCACCACCGGCAAGCTGCCCGTGCCCTGGCCCACCCTCGTGACCACCCTGGGC		Insert for pMMB-*mgtL_mgtA*$_{1\text{-}5aa}$:*yfp*
G081	YFPop-F	TCCAAAATCGTAAAAATCGTGAGC	pMMB-*mgtL_mgtA*$_{1\text{-}5aa}$:*yfp*	Construction pMMB-*mgtL:yfp*
G080	MgtL_YFPop-R	ACGATTTTTACGATTTTGGACCGGAAAAGTTTTAATCTCCGTC		

Supplementary Table 6 (continued)

H024	E2Q_D4N_ mgtL-F	CAACCTAATCCCACGCCTCTC	pMMB-*mgtL:yfp* pMMB-*mgtL_mgtA*$_{1\text{-}5aa}$*:yfp*	Construction pMMB-*mgtL-QN:yfp* pMMB-*mgtL-QN_mgtA*$_{1\text{-}5aa}$*:yfp*
H025	E2Q_D4N_mgtL-R	AGAGGCGTGGGATTAGGTTGCATAT AACCTCCGGTAAGTGAAAAAAG		
J036	D9N_E10Q_aceB-F	AACTGGCTTTCACAAGGCCGTATG	pMMB-*aceB:yfp*	Construction pMMB-*aceB*-NQ*:yfp*
J037	D9N_E10Q_aceB-R	GCCTTGTGAAAGCCAGTTGATTGGT TGTTGTTGCCTGTTCAGTCATC		
J038	NKDEPDyfp-F	AATAAAGATGAACCAGATGTGAGCA AGGGCGAGGAGC	pMMB-*aceB*-NQ*:yfp*	Construction pMMB_UTR$_{aceB}$_MNKDEPD *:yfp*
J039	0aa_MNKDEPDyfp-R	CTGGTTCATCTTTATTCATCGTGCAG CTCCTCGTCATG		
J038	NKDEPDyfp-F	AATAAAGATGAACCAGATGTGAGCA AGGGCGAGGAGC	pMMB-*aceB*-NQ*:yfp*	Construction pMMB_ *aceB*-NQ$_{1\text{-}10aa}$:NKDEPD:*yfp*
J040	10aa_NKDEPDyfp-R	CTGGTTCATCTTTATTTTGATTGGTT GTTGTTGCCTGTTCAGTCATC		
J038	NKDEPDyfp-F	AATAAAGATGAACCAGATGTGAGCA AGGGCGAGGAGC	pMMB-*aceB*-NQ*:yfp*	Construction pMMB_*aceB*-NQ$_{1\text{-}20aa}$:NKDEPD:*yfp*
J041	20aa_NKDEPDyfp-R	CTGGTTCATCTTTATTCTGCTCGCCA TACGGCCTTG		
J038	NKDEDPyfp-F	AATAAAGATGAACCAGATGTGAGCA AGGGCGAGGAGC	pMMB-*aceB*-NQ*:yfp*	Construction pMMB_*aceB*-NQ$_{1\text{-}30aa}$:NKDEPD:*yfp*$_{1\text{-}30aa}$
J042	30aa_NKDEPDyfp-R	CTGGTTCATCTTTATTTACCGCTTCG GCAGTAAGAATTTGC		
J038	NKDEPDyfp-F	AATAAAGATGAACCAGATGTGAGCA AGGGCGAGGAGC	pMMB-*aceB*-NQ*:yfp*	Construction pMMB_*aceB*-NQ$_{1\text{-}40aa}$:NKDEPD:*yfp*
J043	40aa_NKDEPDyfp-R	CTGGTTCATCTTTATTAAAATGCGTC ACCAGCTCAGTCAG		
J034	NKDEPD_yfp-F	AATAAAGATGAACCAGATGTGAGCA AGGGCGAGGAG CTG	pMMB-*yjbJ:yfp*	Construction pMMB_UTR$_{yjbJ}$_MNKDEPD*: yfp*
J035	NKDEPD_yfp-R	CTGGTTCATCTTTATTCATAATCAAG ACCTCATCGTTAGGTTG		
J065	NKDEPD_syn-codon_yfp-F	CGAGCCAGACGTGAGCAAGGGCGA GGAGC	pMMB_UTR$_{yjbJ}$_MNKDEP D*:yfp*	Construction pMMB_UTR$_{yjbJ}$_MNKDEPD$_{syn\text{-}codon}$*:yfp*
J066	NKDEPD_syn-codon_yfp-R	CTCACGTCTGGCTCGTCTTTATTCAT AATCAAGACCTCATCG		
J060	Mutation_motif_in_yfp-F	CTGTTCACCGGGGTGGTGCC	pMMB_*yfp*$_A$ (*yfp* optimized)	Construction pMMB_UTR$_{aceB}$_MVSKGDE *:yfp*
J061	motif_MVSKGDE_yfp-R	CACCCCGGTGAACAGTTCATCTCCT TTACTTACCATATTCTGTTT C		
J060	Mutation_motif_in_yfp-F	CTGTTCACCGGGGTGGTGCC		Construction pMMB_UTR$_{aceB}$_MVSK-DE:*yfp*
J062	motif_MVSKDE_in_yfp-R	CACCCCGGTGAACAGTTCACTTTAC TTACCATATTCTGTTT CCTGTG		
J060	Mutation_motif_in_yfp-F	CTGTTCACCGGGGTGGTGCC		Construction pMMB_UTR$_{aceB}$– MVSKPDE:*yfp*
J063	motif_MVSKPDE_in_yfp-R	CACCCCGGTGAACAGTTCTTCTGGT TTACTTACCATATTCTGTTTCCTG		
J060	Mutation_motif_in_yfp-F	CTGTTCACCGGGGTGGTGCC		Construction pMMB_UTR$_{aceB}$_MVSDPEE *:yfp*
J064	motif_MVSDPEE_in_yfp-R	CACCCCGGTGAACAGTTCTTCTGGA TCACTTACCATATTCTGTTTCCTGTG		

Supplementary Table 7 Oligonucleotides used for Northern blot, IVTA and Toeprinting assay.

Northern blot probes

No.	Name	Sequence (5’→3’)	Target
D072	Probe_yfp_3'_UTR	CAGCCAAGCTTGCATGCCTGCAGGTCGACTCTAGAGGATCCCCGGG	*mgtA-yfp*
C036	Probe_yfp	CTGGACGTAGCCTTCGGGCATGGCGGACTTGAAGAAGTCGTGCTG	All the other *yfp* fusions

***In vitro* transcription and translation**

No.	Name	Sequence (5’→3’)	PCR template	Purpose
H033	T7_pMMB-F	GCGAATTAATACGACTCACTATAGGGA GCGGATAACAAGATACTGAGCACA	pMMB-*aceB*$_{1\text{-}10aa}$:*yfp*<br>pMMB-aceB-NQ$_{1\text{-}10aa}$:*yfp*<br>pMMB-*yjbJ*$_{1\text{-}5aa}$:*yfp*<br>pMMB-*yjbJ*-NQ$_{1\text{-}5aa}$:*yfp*<br>pMMB-*rraB*$_{1\text{-}10aa}$:*yfp*<br>pMMB-*rraB*-QQ$_{1\text{-}10aa}$:*yfp*<br>pMMB_*mgtL*:*yfp*<br>pMMB_*mgtL*-QN:*yfp*	Templates for *in vitro* transcription used in IVTA
P010	After_*yfp*_sequence-R	TGCCTGCAGGTCGACT		
H033	T7_pMMB_F	GCGAATTAATACGACTCACTATAGGGA GCGGATAACAAGATACTGAGCAC	pMMB-*mgtL_mgtA*$_{1\text{-}5aa}$:*yfp*<br>pMMB_*mgtL*-QN_*mgtA*$_{1\text{-}5aa}$:*yfp*	Templates for *in vitro* transcription used in Toeprinting assay
G077	toe_printing_mgtL-R	GGTTATAATGAATTTTGCTTATTCCTG AAGGTAATAAAGCTGAGC		
H033	T7_pMMB_F	GCGAATTAATACGACTCACTATAGGGA GCGGATAACAAGATACTGAGCAC	pMMB-*yjbJ*$_{1\text{-}5aa}$:*yfp* ;<br>pMMB-*yjbJ*-NQ$_{1\text{-}5aa}$:*yfp*<br>pMMB-*rraB*$_{1\text{-}10aa}$:*yfp*<br>pMMB-*rraB*-QQ$_{1\text{-}10aa}$:*yfp*<br>pMMB-*aceB*$_{1\text{-}10aa}$:*yfp*<br>pMMB-aceB-NQ$_{1\text{-}10aa}$:*yfp*	Templates for *in vitro* transcription used in Toeprinting assay
H059	R_toe_printing_hybridat ion in *yfp* sequence	GGTTATAATGAATTTTGCTTATTCGTC GCCGTCCAGCTCG		
J109	TP_NV1_R	Cy5-GGTTATAATGAATTTTGCTTATT	mRNA	Toeprinting assay RT

Supplementary References:

1 Jansson, M. *et al.* High-level production of uniformly (1)(5)N- and (1)(3)C-enriched fusion proteins in *Escherichia coli*. *Journal of biomolecular NMR* **7**, 131-141 (1996). https://doi.org/10.1007/BF00203823

2 Singh, S. *et al.* Cryo-EM studies of the four *E. coli* paralogs establish ABCF proteins as master plumbers of the peptidyl-transferase center of the ribosome. *bioRxiv*, 2023.2006.2015.543498 (2023). https://doi.org/10.1101/2023.06.15.543498

3 Huter, P. *et al.* Structural Basis for Polyproline-Mediated Ribosome Stalling and Rescue by the Translation Elongation Factor EF-P. *Mol Cell* **68**, 515-527 e516 (2017). https://doi.org/10.1016/j.molcel.2017.10.014

4 Meselson, M. & Yuan, R. DNA restriction enzyme from *E. coli*. *Nature* **217**, 1110-1114 (1968). https://doi.org/10.1038/2171110a0

5 Cammack, K. A. & Wade, H. E. The sedimentation behaviour of ribonuclease-active and -inactive ribosomes from bacteria. *Biochem J* **96**, 671-680 (1965). https://doi.org/10.1042/bj0960671

6 Blattner, F. R. *et al.* The Complete Genome Sequence of *Escherichia coli* K-12. *Science* **277**, 1453-1462 (1997). https://doi.org/doi:10.1126/science.277.5331.1453

7 Ousalem, F. *et al.* Comparative genetic, biochemical, and biophysical analyses of the four *E. coli* ABCF paralogs support distinct functions related to mRNA translation. *bioRxiv*, 2023.2006.2011.543863 (2023). https://doi.org/10.1101/2023.06.11.543863

8 St-Pierre, F. *et al.* One-Step Cloning and Chromosomal Integration of DNA. *ACS synthetic biology* **2**, 537-541 (2013). https://doi.org/10.1021/sb400021j

9 Otsuka, Y. *et al.* GenoBase: comprehensive resource database of *Escherichia coli* K-12. *Nucleic Acids Res* **43**, D606-617 (2015). https://doi.org/10.1093/nar/gku1164

10 Baba, T. *et al.* Construction of *Escherichia coli* K-12 in-frame, single-gene knockout mutants: the Keio collection. *Molecular systems biology* **2**, 2006 0008 (2006). https://doi.org/10.1038/msb4100050

11 Datsenko, K. A. & Wanner, B. L. One-step inactivation of chromosomal genes in *Escherichia coli* K-12 using PCR products. *Proc Natl Acad Sci U S A* **97**, 6640-6645 (2000). https://doi.org/10.1073/pnas.120163297

12 Fostier, C. R. *et al.* Regulation of the macrolide resistance ABC-F translation factor MsrD. *Nature Communications* **14**, 3891 (2023). https://doi.org/10.1038/s41467-023-39553-8

13 Furste, J. P. *et al.* Molecular cloning of the plasmid RP4 primase region in a multi-host-range tacP expression vector. *Gene* **48**, 119-131 (1986). https://doi.org/10.1016/0378-1119(86)90358-6

14 Lipońska, A. *et al.* Base composition at the start of the coding sequence controls the balance between translation initiation and mRNA degradation in *E. coli*. *bioRxiv*, 2024.2003.2021.586065 (2024). https://doi.org/10.1101/2024.03.21.586065

15 Boel, G. *et al.* The ABC-F protein EttA gates ribosome entry into the translation elongation cycle. *Nat Struct Mol Biol* **21**, 143-151 (2014). https://doi.org/10.1038/nsmb.2740
