# Supplementary material for: Global regulation via modulation of ribosome pausing by the ABC-F protein EttA
Source: Nat Commun. 2024 Jul 26;15:6314. doi: 10.1038/s41467-024-50627-z (PMC11282234; doi:10.1038/s41467-024-50627-z)

Source Data file

Global regulation via modulation of ribosome pausing by by the ABC-F protein EttA

Farès Ousalem[1§], Saravuth Ngo[1], Thomas Oïffer[1], Amin Omairi-Nasser[1], Marion Hamon[2], Laura Monlezun[1], Grégory Boël[1,*]

[1] Expression Génétique Microbienne, CNRS, Université Paris Cité, Institut de Biologie Physico-Chimique, Paris, France.

[2] Centre National de la Recherche Scientifique (CNRS), Institut de Biologie Physico-Chimique, Plateforme de Protéomique, FR550, F-75005 Paris, France.

[§] Current addresses: Biomarqueurs et nouvelles cibles thérapeutiques en oncologie, INSERM U981, Université Paris Saclay, Institut de Cancérologie Gustave Roussy, 11 rue Edouard Vaillant, 94805 Villejuif Cedex, France.

[*]Correspondence to:
Grégory Boël, Institut de Biologie Physico-Chimique, 13 rue Pierre et Marie Curie, 75005 Paris, France, Tel: +33-1-58415121; e-mail: boel@ibpc.fr

Contents:

Uncropped gel presented in Figures: 3c-d, 5d and in Supplementary Figures: 1a, 2b and 6c.

Excel file with the raw data used for the figures in Main and Supplementary Information.

Uncropped gel

Figure 3c

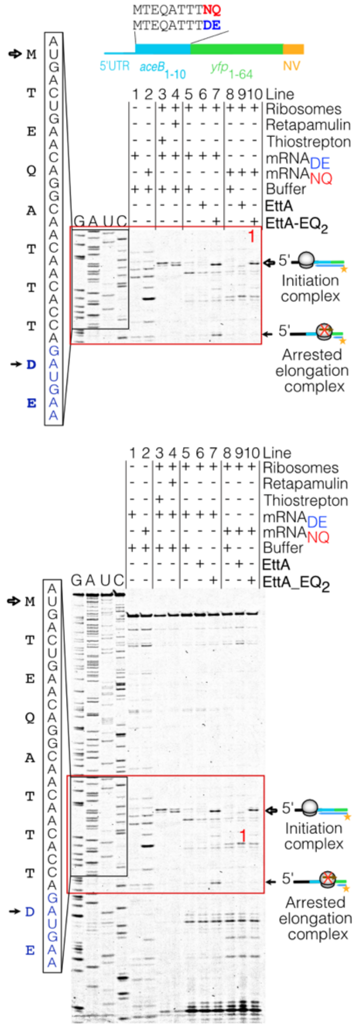

Figure 3d

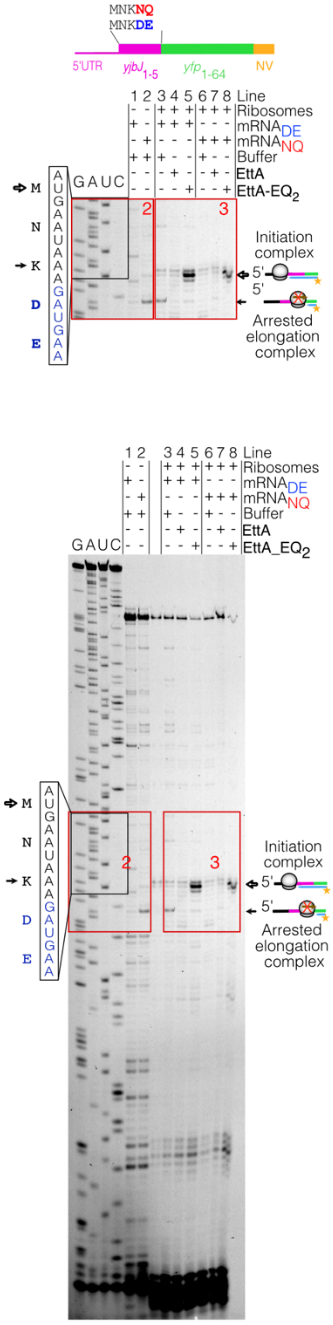

Figure 5d

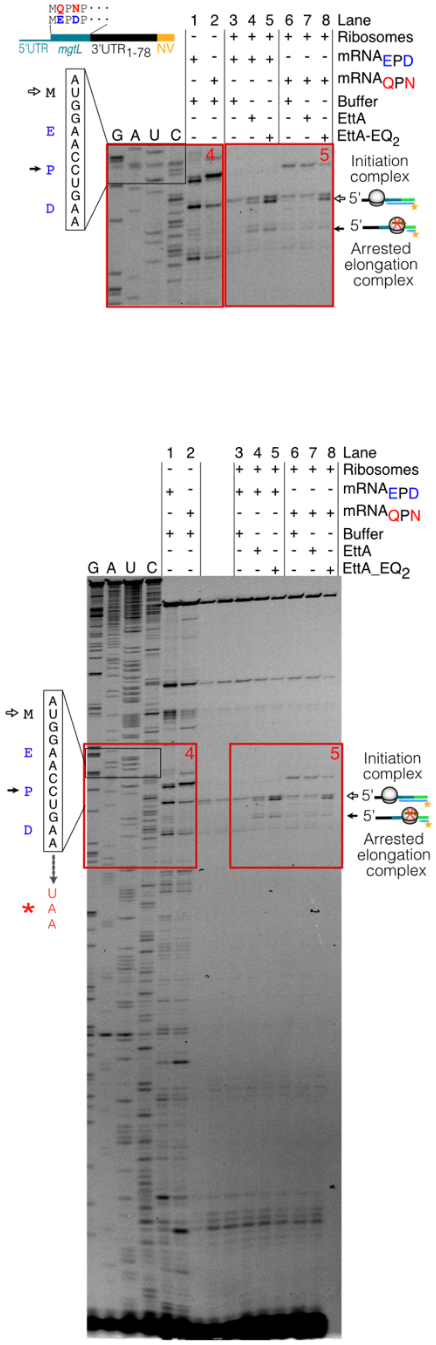

Uncropped gel

Supplementary Figure 1a

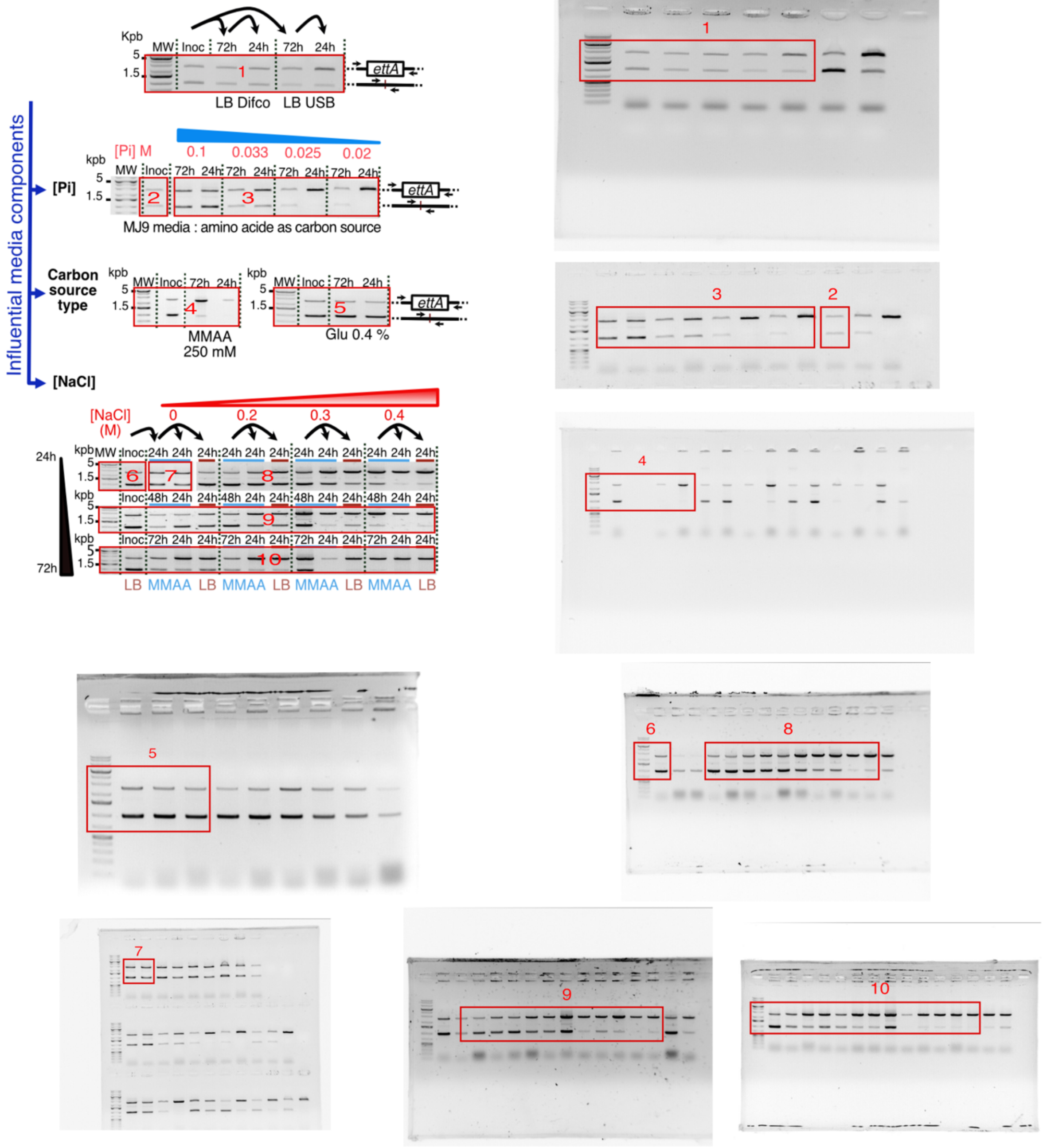

Uncropped gel

Supplementary Figure 2b

Western Blot : *aceA:yfp* and *aceB:yfp* (1)

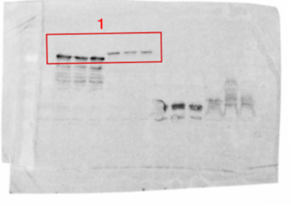

Northern Blot : *aceA:yfp* and *aceB:yfp* (2)

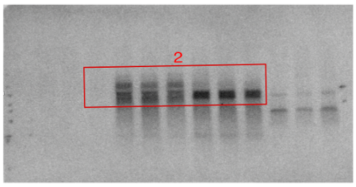

Western Blot : *fumC:yfp* (1)

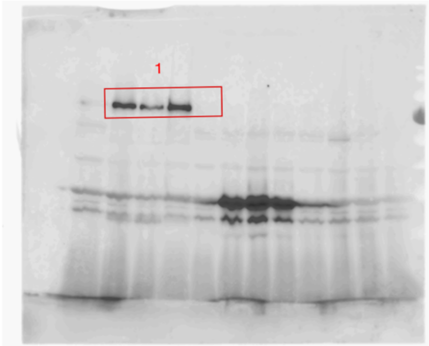

Northern Blot : *fumC:yfp* (2)

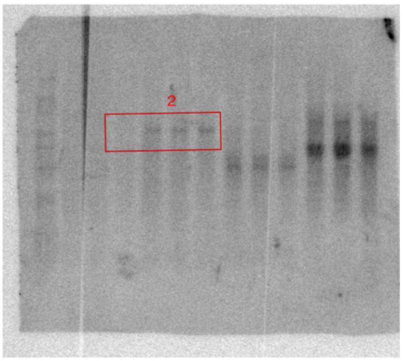

Western Blot : *yjbJ:yfp* (1) and *hchA:yfp* (2)

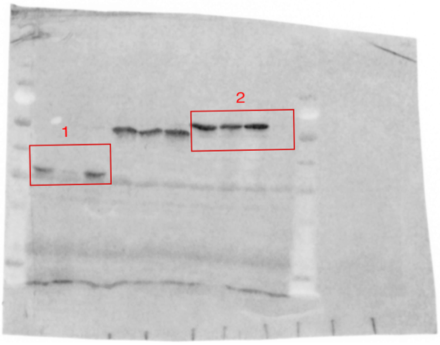

Northern Blot : *yjbJ:yfp* (3) and *hchA:yfp* (4)

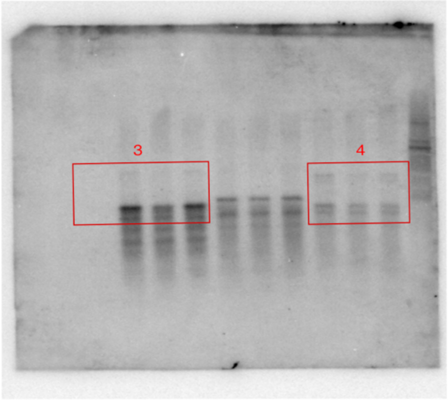

Supplementary Figure 6c

Northern Blot : *mgtL_mgtA*1-5:*yfp*

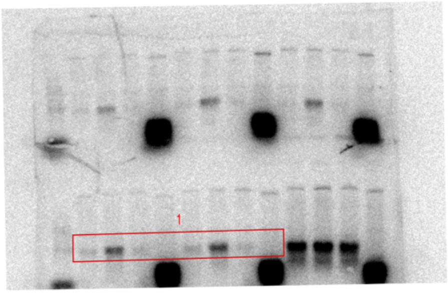

Supplement: Supplementary file 4 — Source Data file [file 41467_2024_50627_MOESM4_ESM.zip › Source_Data/Ousalem_EttA_Source Data file.pdf]
